# Critical Players and Therapeutic Targets in Chronic Itch

**DOI:** 10.3390/ijms23179935

**Published:** 2022-09-01

**Authors:** Hua Yang, Weiwei Chen, Renkai Zhu, Jiafu Wang, Jianghui Meng

**Affiliations:** 1 School of Life Sciences, Henan University, Kaifeng 475001, China; 2 School of Biotechnology, Faculty of Science and Health, Dublin City University, Glasnevin, D09 Dublin, Ireland; 3 National Institute for Cellular Biotechnology, Faculty of Science and Health, Dublin City University, Glasnevin, D09 Dublin, Ireland

**Keywords:** atopic dermatitis (AD), B-type natriuretic peptide (BNP), chronic pruritus (CP), interleukin-31 (IL-31), oncostatin M (OSM), protease-activated receptor 2 (PAR2), T helper 2 (T_H_2), transient receptor potential vanilloid-3 (TRPV3), thymic stromal lymphopoietin (TSLP), Mas-related G-protein-coupled receptors (Mrgprs)

## Abstract

Chronic itch is one of the most prominent clinical characteristics of diverse systematic diseases. It is a devastating sensation in pathological diseases. Despite its importance, there are no FDA-labelled drugs specifically geared toward chronic itch. The associated complex pathogenesis and diverse causes escalate chronic itch to being one of the top challenges in healthcare. Humanized antibodies against IL-13, IL-4, and IL-31 proved effective in treatment of itch-associated atopic dermatitis but remain to be validated in chronic itch. There are still no satisfactory anti-itch therapeutics available toward itch-related neuropeptides including GRP, BNP, SST, CGRP, and SP. The newly identified potential itch targets including OSM, NMB, glutamate, periostin, and Serpin E1 have opened new avenues for therapeutic development. Proof-of-principle studies have been successfully performed on antagonists against these proteins and their receptors in itch treatment in animal models. Their translational interventions in humans need to be evaluated. It is of great importance to summarize and compare the newly emerging knowledge on chronic itch and its pathways to promote the development of novel anti-itch therapeutics. The goal of this review is to analyze the different physiologies and pathophysiologies of itch mediators, whilst assessing their suitability as new targets and discussing future therapeutic development.

## 1. Introduction

Chronic pruritus (CP) or chronic itch is a devastating clinical condition that entails continuous itch for more than 6 weeks, but there are limited medications to help with this condition. The complexity and heterogeneity of this clinical condition pose a major challenge for clinical care. Genetic studies and single-cell sequencing data have provided in-depth neural–immune networks encoding itch information in different mechanisms of itch. The detailed signature of itch-sensing neurons has been characterized but is still largely unknown. There are no therapeutics that are generally effective. Dysregulated communications between sensory and immune cells, skin resident cells, as well as central systems trigger chronic itch and chronification. When the skin is irritated, itch signals are sent to the peripheral nerve of the cutaneous nerve endings, which originated from dorsal root ganglion (DRG), then are ascended to the spinothalamic tract and the parabrachial nucleus at the other side before reaching the somatosensory thalamus, and then are projected on the cortex [1,2,3,4]. Diverse chemical stimuli trigger itch, including neuropeptides [5,6,7,8,9,10], amines [11,12], cytokines [13,14,15,16,17,18,19], chemokines [20,21], proteases [22,23,24,25], lipids [26,27,28,29], and opioids [30]. The goal intended to be achieved by this review is to understand the different physiologies and pathophysiologies of itch and discuss potential emerging targets as well as achievements in ongoing therapeutic development.

## 2. Neuropeptide Targets in Itch Transmission

Neuropeptides are critical in the transmission of itch sensation from the peripheral nervous system to the spinal nervous system and to higher levels. Diverse neuropeptides including calcitonin gene-related peptide (CGRP), substance P (SP), and vasoactive intestinal peptide (VIP) can promote skin inflammation [6,31,32]. B-type natriuretic peptide (BNP) and gastrin-releasing peptide (GRP) [4] play critical itch-selective roles in itch sensation [33]. The inter-neurons are defined by the neurochemical and neuropeptidergic characteristics [34], based on marker molecule expressions including somatostatin (SST), neuropeptide Y (NPY), and parvalbumin (PV) [35,36].

### 2.1. CGRP

CGRP is an extensively studied vasodilator that is expressed in the sensory neurons, motor neurons, and many other cell types, including monocytes/macrophages [37], Langerhans cells (LCs, dendritic antigen-presenting cells that reside within the epidermis) [38] and keratinocytes [39], but not in basophils. Its splice variants, CGRPα is mainly found in the central nervous system (CNS) and peripheral nervous system (PNS), whereas CGRPβ is less expressed in PNS but is the only isoform in keratinocytes [40]. Activation of *TRPV1*^+^ sensory neurons release CGRP to act on diverse immunocytes, including T cells, B cells, dendritic cells (DCs), mast cells, macrophages, and LCs, causing neuro-inflammation, neurogenic vasodilation, and immune response [41]. CGRP released from other cells (i.e., cutaneous lymphocyte-associated antigen (*CLA*)^+^ T cells) may exaggerate neurogenic inflammation response and induce the release of itch-related cytokines, i.e., interleukin-4 (IL-4) or interleukin-13 (IL-13) from immune cells [42]. CGRP was involved in the regulation of pruritus. Significantly higher CGRP concentration was detected in patients suffering from severe pruritus [43]. The plasma CGRP level of patients with greater eczema score (SCORing Atopic Dermatitis, SCORAD) was significantly higher than that of patients with lower SCORAD score. *CGRP*^+^ interneurons mediate spinal itch transmission but not pain signals [33]. Although the deletion of *CGRPα*^+^ sensory neurons in mice showed a reduced sensitivity to histamine and chloroquine-induced itch [44], intradermal injection of CGRP failed to induce skin itch response and did not cause itch in humans [45]. An antagonist for CGRP has proved effective in relief of pain and migraine [46], and also reduces itching caused by harmful heat stimuli, histamine, and chloroquine [44]. The prospective for the usage of CGRP antagonists in treatment of itch in humans still remains elusive.

### 2.2. SP

SP is an important transmitter of the afferent neurons in the PNS and CNS. SP acts on diverse immunocytes, including eosinophils, mast cells, and T cells, and promotes skin inflammation [47,48]. Although neurokinin-1 receptor (NK-1R) has been traditionally regarded as the main SP receptor, mas-related G-protein-coupled receptor member B2 (MrgprB2)/MrgprX2) on the mast cells may be the critical receptor mediating SP-mediated mast cell activation [49]. The SP-activated mast cells release histamine, leukotriene B4 (LTB4), prostaglandin D2 (PGD2), and tumor necrosis factor alpha (TNF-α) [50,51,52] to further induce SP release from the sensory nerve endings and exacerbate itch. SP may trigger the release of pruritogenic compounds from other cell types such as keratinocytes and endothelial cells [53,54]. SP is also involved in the transmission of itch sensation at the spinal cord level [9]. In contrast to CGRP, intradermal injection of SP into the mouse dose-dependently produced scratching at the injected site and peaked 10 min after injection [55]. An antagonist for NK-1R, aprepitant, has proved beneficial in itch relief in phase II clinical studies [56]; however, the safety and long-term effect is unclear.

### 2.3. BNP

**Figure 1:**
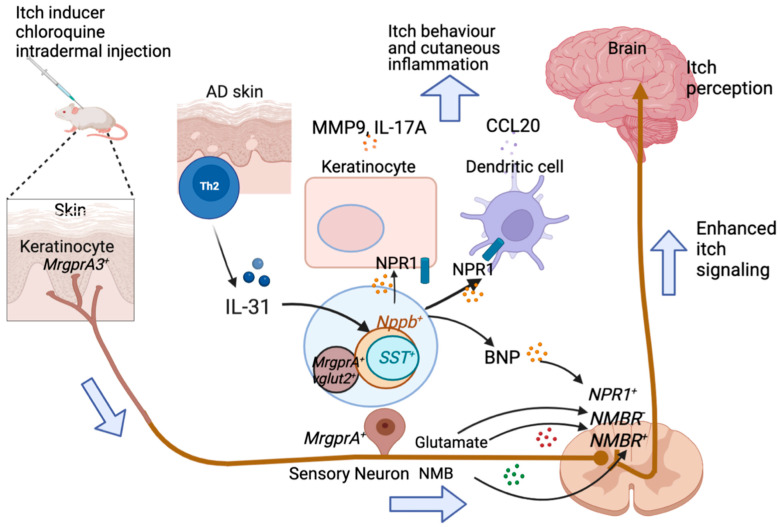
Glutamate and BNP transmit itch through distinct pathways. *MrgprA3*^+^ neurons utilize glutamate together with NMB, to transmit itch signal to dorsal horn neurons. Distant from glutamate that activates *NMBR*^+^ and *NMBR*^-^ dorsal horn neurons, NMB activates only *NMBR*^+^ neurons. Instead, BNP is expressed in a separate neuronal subpopulation which also express SST and IL-31RA. During AD itch, IL-31 induces BNP release from these *Nppb*^+^ neurons, and the released BNP binds *NPR1*^+^ dorsal horn neurons to propagate itch signal.

BNP is a central itch mediator [5]. Release and synthesis of BNP is upregulated by interleukin-31 (IL-31) in sensory dorsal root ganglionic neurons (DRGs) [18,57] (Figure 1). IL-31 receptors (IL-31RA and OSMR) are co-enriched with the BNP gene (Nppb) in DRG [58,59]. BNP and receptor expression is increased in the pathogenic skin of AD patients [58,59]. In the human skin cells, the pro-inflammatory and itch-promoting phenotypes are promoted by BNP [18]. Moreover, the itch responses to subcutaneously injected IL-31 were significantly attenuated in Nppb-KO mice and mice treated with Nppb-saporin, a toxin that ablated 70% of BNP receptor-positive neurons in the spinal cord [60,61]. BNP was found to significantly promote neuromedin B (NMB)-mediated scratching behavior [62]. Nppb saponin simultaneously ablates spinal cord Npr1 and Npr3 neurons, impairing histamine- but not CQ-induced pruritus [62]. In the skin, BNP was found to sensitize transient receptor potential vanilloid 3 (TRPV3), resulting in enhanced Serpin E1 release, an itch-specific mediator with transcription levels positively correlated with the severity of human AD skin [63]. Thus far, no BNP or NPR1 antagonist has been identified for itch relief in humans, although the NPR1/2/3 antagonist is effective in chronic itch models [64]. In AD-like murine models, an antagonist for Serpin E1 reduced itch-like behaviors [63], revealing underlying potential for antagonizing Serpin E1 in itch relief.

### 2.4. GRP

GRP is a spinal itch-selective transmitter known to be highly expressed in a population of spinal cord dorsal horn (DH) interneurons [33,65,66,67], and may serve as a “leaky gate” for nociceptive signals [68]. GRP18-27 induces scratching behavior upon intrathecal injection via activation of the GRP receptor (GRPR) [69]. Spinally restricted ablation of GRP neurons reduced itch-related behaviors to different pruritogens, whereas their chemogenetic excitation elicited itch-like behaviors and facilitated responses to several pruritogens. In contrast, responses to painful stimuli remained unaltered. GRP neurons receive direct input from *MrgprA3*^+^ pruritoceptors [33]. Since glutamate and NMB are implicated in *MrgprA3*^+^ neurons, how the itch signal is coded with GRP is not clear. These data confirm a critical role of dorsal horn GRP neurons in spinal itch transmission [33]. GRP level is also increased in AD patient skin and GRP seems to promote thymic stromal lymphopoietin (TSLP) release from keratinocytes [70]. Despite its pivotal role in regulating itch, therapeutic interventions in GRP signaling have not been progressed so far, mainly due to a complex integration of central circuits within the spinal cord level and a variety of modulators involved. Apart from this, it is reported that intradermal injection of GRP in mice also elicited scratching, in addition to mast cell degranulation [8]. It remains unknown if targeting GRP peripheral signaling would be beneficial for clinical treatment of itch.

### 2.5. NMB

Both NMB and GRP are related to the amphibian bombesin protein [71], and seem to act as itch-specific neuropeptides in DRGs [72]. Intrathecal injection of NMB elicits scratching behavior, similar to GRP, at a picomolar dose range [73], but intradermal injection of NMB also induces itch at a nanomolar dose range [74]. NMB and GRP encode predominantly histaminergic pruritus and non-histaminergic pruritus, respectively [73,75]. Each of them can bind to the cognate receptor GRPR and NMB receptor (NMBR) in the spinal cord. Moreover, the NMB is wide-spread in sensory neurons, and together with glutamate is highly expressed in *MrgprA3*^+^ neurons, which form monosynaptic, glutamatergic connections with *NMBR*^+^ and *NMBR*^−^ DH neurons and function in a context-dependent manner (pruritogen type, concentration, etc.) to enhance the activity of *NMB*R^+^ DH neurons [76]. Glutamate is an essential itch mediator in sensory neurons expressing MrgprA3/NMB/Vglut2, and its signaling to *NMBR*^+^ DH neurons is enhanced by NMB from *MrgprA3*^+^ afferents [76]. *MrgprA3*^+^ and *NPPB*^+^-neurons are in distinct neuronal subsets, and each are labelled by different itch biomarkers and receptors (Figure 1). Interestingly, BNP is found to crosstalk with NMB in the spinal cord by facilitating NMB-involved itch through each respective receptor NPR3-NMBR co-signaling [62]. How this signaling is manipulated by GRP and glutamate requires further investigation. The antagonist for NMB signaling (CNQX, i.t. injection) blocks chloroquine-induced itch in mice [73]. However, its potential as a therapeutic target in chronic itch at both peripheral and central level is not clear.

### 2.6. SST

SST originates from the DRGs and spinal dorsal horn neurons and is an important endocrine hormone and a neuropeptide in the nervous system, including the PNS and CNS. SST induces scratching behavior in rats when intrathecally injected [77]. It is not known yet whether intradermal injection of SST induces peripheral itch in mammals, despite that SST is expressed in *Nppb*^+^ DRGs (Figure 1), and optogenetically activated *SST*^+^ primary afferent neurons triggered itch behavior [58]. In addition, *SST*^+^/*Nppb*^+^ neurons mediate mast-cell-induced itch [78,79]. At the spinal cord level, spinal *SST*^+^ neurons partially overlapped with *NPR1*^+^ neurons, the receptor of BNP [80]. The transcription factor *Bhlhb5*^+^ neurons that express the antipruritic neuropeptide endorphin were found to be hyperpolarized by SST, resulting in disinhibition of *GRPR*^+^ neurons, underlying the SST potentiation of chemically induced scratching [58]. However, how SST feeds back to BNP signaling from the spinal to peripheral system has not been clarified.

### 2.7. Endothelin-1 (ET-1)

ET-1 is a neuropeptide in spinal cord and sensory neurons [81], and it is also released by the keratinocytes, endothelial cells, immunocytes, and neurocytes. ET-1 is an effective vasoconstrictor related to itch sensation in humans and is one of the most effective itch sources that directly act on the sensory neurons [82,83,84,85,86,87,88]. It binds with the endothelin A receptor (ETAR) and endothelin B receptor (ETBR), inducing strong itch response on mammals as a non-histamine itch agent, and mediates a histamine-dependent itch reaction in humans. Neural peptidase endothelin-converting enzyme 1 (ECE-1) regulates the ET-1-induced scratching reaction in mice [82,86]. Moreover, both the epidermal level and plasma level of ET-1 are increased in human AD, and ET-1 also induces BNP release from sensory neurons, in a similar manner as IL-31 [89], underlying the exacerbated atopic itch and associated dysregulated vascular reactions in human skin. Topical application of its dual receptor antagonist bosentan effectively relieved mite-induced AD-like itch [90]. ET-1 also plays critical roles in psoriasis and Prurigo Nodularis, and ETAR antagonist ambrisentan improved the symptom in psoriasiform dermatitis in a mouse model [91], highlighting their possible anti-pruritic potential by antagonizing ET-1 pathway.

## 3. Itch Targets in G-Protein-Coupled Receptor Pathways

GPCR detects diverse itch-inducing chemical substances. Many types of GPCR have been discovered, such as histamine-related H1 and H4 receptors, protease-activated receptor 2 (PAR2), Mrgpr, IL-31RA, IL-13RA, IL-4R, CysLT receptors, TSLP, and endothelin receptors, etc. [23,92,93]. The G-protein-coupled signal cascade is triggered via phospholipase C and phospholipase Gβγ, and thus mediates TRP cation channel gating [88,94].

### 3.1. Histamine

Histamine is a common inflammatory mediator and the most known itch inducer. It is released mainly by the mast cells and basophils and occasionally by keratinocytes. It activates eosinophils, mast cells, basophils, and T_H_2 cells [3,95,96,97,98,99,100]. Among the known four histamine receptors, H1, H2, H3, and H4, histamine induces the itch sensation via activation of H1 and H4 receptors on sensory neurons [11], resulting in activation of TRPV1 via phospholipase, leading to the release of neuropeptides such as CGRP and SP, thus inducing neurogenic inflammation, local vasodilation, plasma extravasation, and mast cell degranulation [3,95,96]. Spinal cord H4R-mediated itch can be persistent, and antagonists for H4R attenuated itch in AD patients whereas antagonists for H1R and H2R are largely ineffective in AD and psoriasis [101,102]. However, although the antagonists of H4R are tested in clinical trials, there is not even one that meets the clinical standard for chronic itch. Oral ZPL-3893787 improved inflammatory skin lesions in patients with AD; however, this still requires further investigation to give a conclusive result in the reduction in pruritus [103].

### 3.2. Proteases, Tryptase, and Kallikreins (KLK) 5 and 14

Specific serine and cysteine proteases, including tryptase, KLK 5 and 14, and cathepsin activate PAR2, which plays a critical role in itch sensation and cutaneous inflammation in mice and humans [104]. Intradermal injection of tryptase activates PAR2 to elicit scratching in mice [25]. Tethered ligands, such as SLIGRL (agonist of PAR2) and AYPGKF (PAR4 agonist), elicit non-histamine-dependent scratching bouts in mice, but not rats [105]. Moreover, cathepsin S cysteine protease cleaves PAR2 and PAR4 as well as MrgprC11 to produce itch in mice [23]. PAR2 activation induces mobilization of keratinocytes and TSLP through phospholipase C (PLC), and this itch-related process is regulated by the PAR2/TRPV3 signaling cascade in keratinocytes [106]. TRPV3 and PAR2 are upregulated in skin biopsies of patients with AD and mice, while in mouse models of AD, their inhibition attenuates itch-related behaviors and inflammation. In a murine model of AD, pruritus, local skin inflammation, and epidermal thickening were significantly inhibited by a PAR2 antagonist, PZ-235 [107]. Whether the PAR2 antagonist can improve debilitating itch and skin lesions in AD without causing immune suppressive side effects is still a challenging unmet clinical need.

### 3.3. Mas-Related G-Protein Coupled Receptors (Mrgprs)

Among 50 mouse and 10 human Mrgpr subjects, mouse MrgprA1, A3, B2, C11, and D, and human X1, X2, and X4, are characterized as itch receptors. Mouse *MrgprA1*^+^ sensory fibers respond to bilirubin and SP to induce itch [108]. *MrgprA3*^+^ and its human ortholog MRGPRX1 respond to CQ, BAM8-22, and mucunain [76]. Mouse MrgprB2 is homologous to human MRGPRX2 in mast cells, except for the absence of MrgprB2 from nerves [109,110,111,112,113]. Tick salivary peptides induce itch through MrgprC11/MRGPRX1 signaling in sensory neurons and also promote acute skin inflammation via mouse MrgprB2 and human MRGPRX2 on mast cells [114]. Mouse *MrgprD*^+^ neurons innervating the epidermis sense itch, and nonpeptidergic *MrgprD*^+^ neurons are implicated in immunoregulatory function and cutaneous immune homeostasis [115]. Mouse MrgprA3^+^ account for 95.2% of *MrgprC11*^+^ neurons, and *MrgprD*^+^ account for 18.5% *MrgprC11*^+^ neurons; however, these have distinct roles, particularly in glabrous skin itch, which is sensed mainly by MrgprC11 [79]. SP also activates mouse MrgprB2 and human MRGPRX2 to induce mast cell degranulation and drives allergic contact dermatitis and non-histaminergic itch in mice [116]. Mouse MrgprC11 also responds to BAM8-22, Cathepsin S, and SLIGRL. In both human and rodents, B-alanine activates MrgprD to induce itch [117]. In humans, MrgprD is co-localized with MRPGRX1 in the *TRPV1*^+^ sub-population in DRG. In addition, like mouse MrgprA1, sensory neuronal MRGPRX4 in humans is activated by bilirubin to sense cholestatic itch [118]; however, distant from MRGPRX4 that can be activated by bile acids, none of mouse Mrgprs respond to it [118,119]. MRGPRX3 is overexpressed in AD [120], however, its ligand and relation to itch remain elusive. Currently, new antagonists for inhibiting MRGPRX4 (EP547) are under clinical development by Escient Pharmaceuticals, with a positive effect in phase I for safety, tolerability, and pharmacokinetic (PK) profile, and great potential treatment of cholestatic and uremic pruritus (ClinicalTrials.gov Identifier: NCT04510090).

### 3.4. 5-Hydroxytryptamine (5-HT, Serotonin)

The neurotransmitter 5-HT is important in the CNS and is an inflammatory mediator released by the mast cells, melanocytes, and platelets [121]. In addition, 5-HT is required for both central and peripheral itch sensation, its skin and spinal cord levels are increased in chronic itch models, and it is able to induce itch upon intradermal injection in mice [122,123], in rats [124], and in humans [125]. Serotonin-triggered acute itch requires both HTR7 and TRPA1, and AD itch also requires HTR7 [126]. Mice lacking HTR7 or TRPA1 displayed reduced scratching and skin lesion severity in a mouse model of AD [126]. In addition, 5-HT- and glucosyl sphingosine-induced itch also involve sensory neuronal TRPV4 and 5-HT2 receptors [127,128]. How these two distinct pathways cooperate in different itch conditions is unclear. Central 5-HT1A descends signals through GRP-GRPR pathways to promote itch output [129]. Targeting 5-HT receptors may be beneficial for relief of itch in cholestatic condition [130], in AD [128], and psoriasis patients associated with anxiety [131]; however, apart from several antagonists being effective in mice models, no 5-HT targeted therapeutics are available for clinical treatment of itch.

### 3.5. Leukotrienes

The leukotrienes are eicosanoids which are synthesized from arachidonic acid under the catalyst of lipoxygenase (LO). Leukotriene C4 (LTC4) and LTB4, but not LTD4 and LTE4, induce itch. LTC4 activates peripheral neuronal CysLT2R to induce acute itch, and LTC4 is implicated in the MC903-induced AD-like itch, but not dry skin itch [132]. LTB4 induces scratching behavior in mice [133,134,135], and was increased in the AD lesional skin of patients and in an AD mouse model [27]. Antigen-induced activation of basophils release LTC4 to activate sensory neuronal CysLT2R to induce acute itch flares [136]. SP-induced scratching may be partially mediated by LTB4 released from the keratinocytes, and LTB4 release can be mediated by several stimuli, and thus drives neutrophil recruitment to the skin [137]. Sphingosyl phosphoryl choline (SPC)-induced scratching may be associated with LTB4 synthesis and released by the keratinocytes [73]. LTB4 receptor 1 (BLT1) and BLT2 are the two receptors for LTB4, and these are expressed in peripheral sensory neurons with opposing roles in sensitization [138]. BLT2 in neutrophils is known to mediate LTB4-induced allergic skin inflammation [139]. Among the LTB4 antagonists, Etalocib, Amelubant, and Moxilubant have been actively developed in clinic trials, yet none have been indicated for anti-itch uses.

## 4. Cytokine and Chemotactic Factor Induced Itch

The cytokines build “a bridge of communication” between the immune system and the nervous system. AD-related skin lesions and itch are aggravated under the mutual interaction of neural–epidermal immune signal pathways. Pruritus is caused by a variety of pruritus-derived cytokines, including TSLP, interleukin-2 (IL-2), interleukin-4 (IL-4), interleukin-13 (IL-13), IL-31, interleukin-33 (IL-33), etc. [140,141,142,143,144], and by the imbalance of the neuro–immune circuit between the receptors IL-4R, IL-13R, IL-31RA, OSMR, Mrgprs, and itching peptides (SP, BNP, CGRP, GRP and protease, etc.) [145,146]. As the quantity of T_H_2 cells is increased, the inflammation related to specific cytokines and the generation of eosinophilia and immunoglobulin E (IgE) are promoted, whilst the generation of epidermal barrier proteins and antibacterial peptides is inhibited. IL-4 and IL-13 are typical type 2 cytokines and have been proven to directly stimulate the sensory neurons via the Janus Kinase 1 (JAK1) signals and promote itch sensation, and IL-5 is considered as a critical link in the growth, differentiation, and migration of the eosinophils and the recruitment of the eosinophils.

### 4.1. IL-13

**Figure 2:**
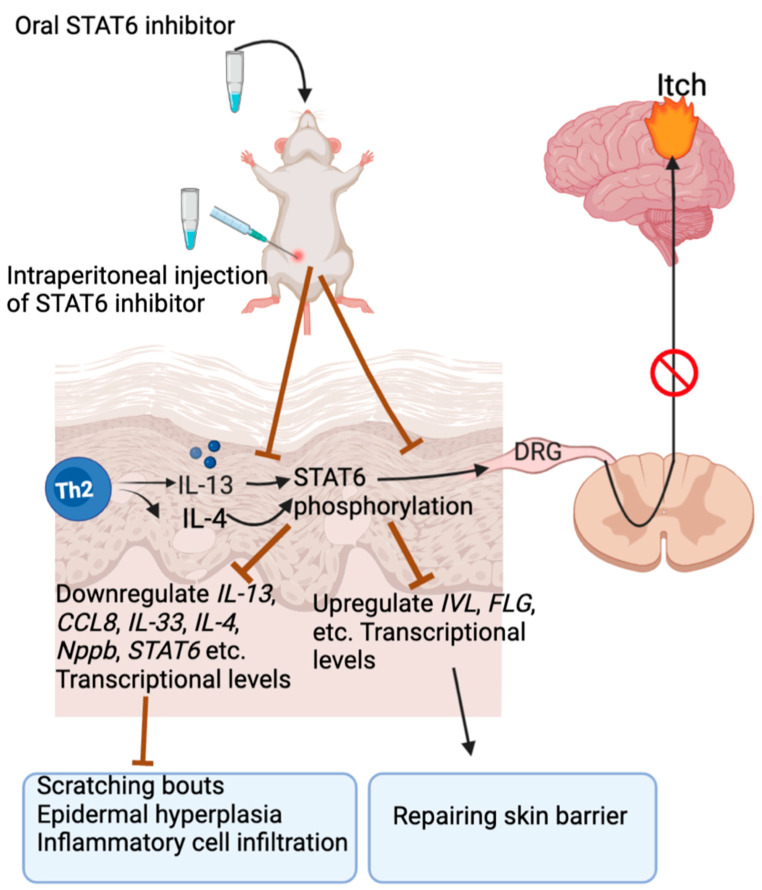
STAT6 mediates IL-13-induced itch signaling. IL-4 induces STAT6 activation in keratinocytes, and IL-13 triggers STAT6 activation to upregulate periostin and IL-24 expression, therefore both promoting skin barrier dysfunction via downregulation of filaggrin. STAT6 Y641 phosphorylation is upregulated in patient skins with AD. Pharmacological inhibition of STAT6 Y641 phosphorylation attenuates MC903-induced ear thickening, immune cell infiltration, and transcription of *IL-13*, *IL-4*, and *CCL8*, while promotes transcripts involved in maintaining skin barrier. Both intraperitoneal and orally active inhibitors of STAT Y641 (namely AS1517499 and AS1810722) are effective in prevention of MC903-incuded itch and cutaneous inflammation.

IL-13 levels are increased in skin and serum from AD patients, and IL-13 participates in the initiation of AD and itching. Together with IL-4, it aggravates epidermal barrier dysfunction by downregulation of the filaggrin (FLG) and involucrin (IVL) expression in the keratinocytes [147,148]. The sensory neurons and keratinocytes express heterodimer receptor IL-4, receptor alpha/IL-13, receptor alpha 1 (IL-4Rα/IL-13Rα1), and IL-13 receptor alpha 2 (IL-13Rα2) [19,149]. IL-13 binds with IL-13Rα1 with low affinity, and when the heterodimer receptor consisting of IL-13Rα1 and IL-4Rα is formed, with high efficiency, the latter is a type II receptor [150]. IL-13 activates sensory neurons directly, however, it may not induce itch directly in mice, but acts as an enhancer of other stimuli, such as histamine [17]. IL-13 and TLR2 heterodimer agonists can upregulate the transcription of IL-13Rα2 in keratinocytes and sensory neurons, respectively [149], thereby promoting neurogenic inflammation and exacerbating AD and itch. Breaking the connection between cutaneous IL-13 and IL-13Rα2 as well as TLR2 and IL-13Rα2 will block the excessive release of these cytokines and hypersensitization of AD, and therefore attenuate itching conditions [149]. Interestingly, Lebrikizumab, a mouse antibody that interacts with IL-13 and is effective in treatment of AD itch, reduces neuronal gene transcription in human sensory neurons, including *IL-13Rα2*, suggestive that IL-13Rα2 is a downstream target and contributes to the relief of AD itch [144]. STAT6 has been identified as a prime target in IL-13-mediated itch and skin inflammation in murine AD (Figure 2). STAT6 selective inhibitors are proved effective in attenuating scratching bouts, downregulation of transcriptional levels of a various of itch-related mediators, and rescuing skin barrier repairment gene transcription [151].

### 4.2. IL-31

IL-31 plays an important role in the induction of itch and inflammation in AD and chronic contact dermatitis in mice and humans [18,152,153]. IL-31 stimulates itch-related neuronal subset NP3, a subpopulation also responsive to mast-cell-released 5-HT, leukotriene C4 (LTC4), and S1p [78], and release BNP and SST. Moreover, IL-31 binds to its receptors on epidermal keratinocytes and immune cells (i.e., eosinophils) to induce skin barrier dysfunction and cutaneous inflammation (Figure 1). Compared with IL-31RA, shared subunit gene IL-4Rα of the IL-4 and IL-13 receptors is seemingly widely expressed in itch sensation neuronal subsets NP1, NP2, and NP3, supporting more extensive functions. IL-31RA expression level is increased in AD lesional skin and abundant in the sensory neuronal outgrowth [154]. IL-31 can be induced by IL-33, and thus itch sensation and scratching behavior are promoted [155]. Moreover, the IL-31-mediated sensory sensation is also amplified by IL-4 and IL-13 [156]. Moreover, IL-31 induces *Nppb* transcription from both murine and human sensory neurons [18,144], further exacerbating itch signaling. Dupilumab has proved effective in relief of Prurigo Nodularis and chronic spontaneous urticaria (CSU) in a phase 3 clinical trial, in addition to its indication in AD.

### 4.3. IL-33

IL-33 is an effective amplifier of type 2 immune reaction and is an important target for dry skin pruritus and chronic pruritus of unknown origin (CPUO). IL-33 receptor ST2 (also named IL-33R) is expressed in DRGs [157], keratinocytes, immune cells, fibroblasts, and mast cells [158]. Though plasma levels of IL-33 were elevated in AD patients, the neuronal restricted IL-33R signaling was dispensable for itch in AD-like disease in mice models [143]. The neuron-restrictive IL-33R signals are critical regulatory factors of itch sensation, which are shown in the dry skin environment, and are independent from the immune cells. IL-33 directly stimulates sensory neurons, but its intradermal injection alone does not induce acute itch behaviors, though it can potentiate sensory neurons [159,160]. However, injection of IL-33 into urushiol-challenged skin rapidly exacerbated itch-related scratching via ST2, in a histamine-independent manner [160]. Binding of IL-33 to keratinocytes contributes to the impeded filaggrin and claudin-1 protein expressions and functional damages to the skin barrier, and facilitation of immune regulation. IL-33 stimulates diverse cells including ILC2 and generates type 2 cytokines including IL-5 and IL-13. ILC2 separated from the lesion site is activated by IL-33 rather than TSLP [161].

### 4.4. IL-6

IL-6 is predominantly expressed in dendritic cells, keratinocytes, macrophages, and neurons. The dendritic-cell-derived IL-6 level is linked to AD. IL-6 facilitates production of IL-4 expression by CD4^+^ T cells and their differentiation to T_H_2 cells [162,163]. It was upregulated in the DRG of mice in a contact dermatitis model (a model induced by Diphenylcyclopropenone (DCP)) [164,165,166]. Skin-derived IL-6 is upregulated upon intradermal injection of calcium phosphate (CaP), which mediates mild to severe scratching in mice [167]. IL-6-induced inositol 1,4,5-triphosphate receptor (IP3R1)/transient receptor potential cation (TRPC)-channel-mediated Ca^2+^ signals in the astrocytes are necessary for the continuous signal transducer and activator of transcription (STAT3) activation, LCN2 expression, and chronic itch [164,165,166]. However, the mAb tocilizumab-antagonizing IL-6 receptor was effective in severe AD but associated with bacterial superinfection/immunodeficiency [168,169].

### 4.5. IL-2

IL-2 is an itch inducer as well as an autocrine cytokine, and its single intradermal injection induces a long-time low-intensity local skin itch that lasts 48–72 h, as well as erythema in human AD patients and healthy subjects [170]. Serum levels of its receptor IL-2R are elevated and correlated with the severity in patients with AD and psoriasis [171]. IL-2 is released from keratinocytes and various immune cells, then activates histaminergic neurons. Moreover, it induces erythema and dermal T-cell infiltration [170]. Therapeutics specifically targeting IL-2 or its receptor have not been developed for chronic itch relief; however, gabapentin has been proposed as a treatment option in IL-2-related pruritus, but this requires final investigation at the clinic level [172].

### 4.6. TSLP

TSLP is a pro-allergic cytokine that is mainly released from keratinocytes, and is the prime target in AD. TSLP drives T_H_2-mediated inflammation and enhances periostin release from keratinocytes, thereby promoting itch signaling, and this effect is susceptible to JAK2 inhibitor SD1008 and the STAT3 inhibitor niclosamide [173]. Upon release from keratinocytes, TSLP activates various immune cells such as T cells, dendritic cells, mast cells [174], and sensory neurons directly to evoke itch behaviors [16]. Biological functions of TSLP require heterodimer formation between the TSLP receptor (TSLPR) and interleukin-7 receptor-alpha (IL-7Ra) [175]. Intradermal injection of TSLP into mouse cheek skin induces scratching behavior in a primary afferent neuron-dependent manner together with T cell and eosinophil-dependent systemic T_H_2 inflammatory response [176]. TSLPR activation of primary afferent sensory neurons requires TRPA1 but not TRPV1 [16]. However, the anti-TSLP human monoclonal antibody tezepelumab is modest or nonsignificant compared to placebo in improving pruritic skin lesion in AD, as shown in a randomized phase 2a clinical trial [177,178]. Apart from this, TSLP is also important for promoting wound-induced hair growth and regeneration in mice [179], which may be an issue that should be considered to use TSLP antagonists for pruritus accompanied by hair loss.

### 4.7. Periostin

**Figure 3:**
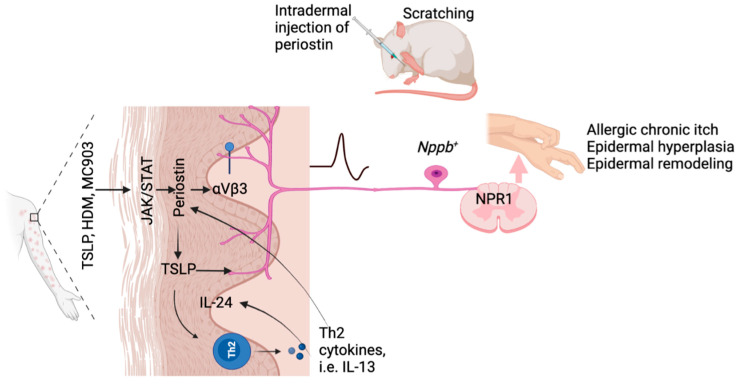
TSLP–periostin–TSLP–NPPB loop in peripheral itch. Periostin is abundantly expressed in the skin of AD patients. TSLP, HDM, and MC903 induce periostin release in a JAK/STAT-mediated mechanism from mouse keratinocytes. Periostin binds directly with integrin aVβ3 that is expressed on a fraction of *NPPB*^+^ sensory itch fibers to induce itch; meanwhile periostin stimulates immune cells to release other itch mediators including Th2 cytokines, resulting in IL-24 upregulation and epidermal barrier dysfunction in allergic skin inflammation. Periostin also induces TSLP release to promote a periostin-TSLP-Th2 cytokine–periostin feedback loop. Intradermal or intracutaneous or subcutaneous injection of periostin directly induces itch in mice, dogs, and monkeys, and this response in mice can be inhibited by cilengitide.

Periostin plays critical roles in pathogenesis of skin fibrosis, lesional AD, psoriasis, allergic skin inflammation, and Prurigo Nodularis [180,181,182]. It is released from dermal keratinocytes and fibroblasts upon stimulation by T_H_2 cytokines IL-13 and IL-4, then activates integrin aVβ3 on a fraction of *SST*^+^/*NPPB*^+^ sensory itch fibers [173]. Meanwhile, periostin stimulates keratinocytes and immune cells to release various cytokines, including T_H_2 cytokines such as IL-31 [180]. MC903 and house dust mites promote periostin release via a JAK/STAT-mediated mechanism [173]. Periostin also induces TSLP release in a periostin-TSLP-T_H_2 cytokine–periostin feedback loop (Figure 3). Intradermal or intracutaneous or subcutaneous injection of periostin directly induces itch in mice, dogs, and monkeys, and this response in mice can be inhibited by cilengitide [173], a broad integrin inhibitor which has been tested in clinical trials for other diseases such as glioblastoma multiforme, except pruritus.

### 4.8. Lipocalin-2 (LCN2)

LCN2 is a central modulator of chronic itch via a STAT3-dependent mechanism in the astrocytes. It is also released by neutrophils and keratinocytes [183]. However, the involvement of the LCN2 cellular receptor (LCN2-R, SLC22A17) is unclear. The Ca^2+^ influx of the IP3R1-dependent Ca^2+^ reaction in the astrocytes is regulated via TRPC [184,185], and DRG-specific IL-6 knockdown, spinal astrocyte–specific IP3R1 knockdown, and pharmacologic spinal TRPC inhibition attenuated LCN2 expression and chronic itch [164]. The serum level of LCN2 is associated with the severity of itch in patients with psoriasis [186]. The function of LCN2 in peripheral itch and the receptor involved is unknown.

### 4.9. Serpin E1

**Figure 4:**
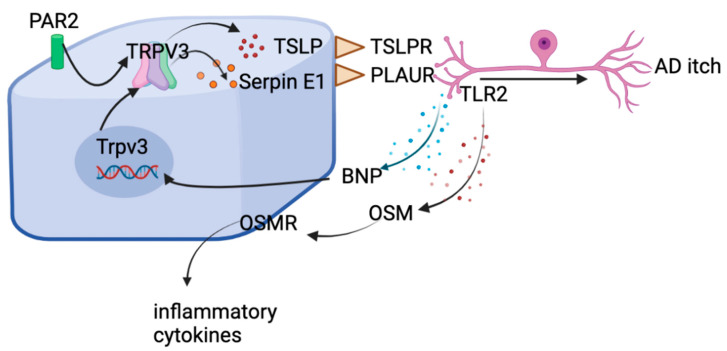
Epidermal PAR2/TRPV3 signaling cascade plays a key role in AD pruritus. PAR2-induced TSLP release requires TRPV3 in keratinocytes and opening of endoplasmic reticulum (ER) Ca^2+^ storage, contributing to acute and AD-related pruritus. In the case of AD, IL-31 induces the synthesis and release of BNP in the neurons, which binds with NPR1 on the keratinocytes to up-regulate *TRPV3* transcription. BNP also enhances the activity of TRPV3 to promote calcium influx and Serpin E1 release. Serpin E1 activates the PLAUR receptor in skin sensory fibers and promotes itch sensation transmission, strengthening the effects of TRPV3-related mediators in dermatitis and pruritus of humans. Serpin E1 receptor PLAUR resides in *TLR2*^+^ neurons and Serpin E1 stimulus leads to transcriptional upregulation of *TLR2* and its co-signaling proteins. The PLAUR-TLR2-OSM signaling promotes skin–nerve communication, cutaneous inflammation, and itch, all feeding into an aggravation of AD and exaggerated itch circuits.

Serpin E1 is produced from neutrophils, glial cells, keratinocytes, and immune cells. Its release from keratinocytes is induced by TRPV3 activation and this effect is enhanced by BNP [63]. IL-31 elevates synthesis and release of BNP in the neurons [18], subsequently upregulating the expression and surface of TRPV3 expression, causing increased TRPV3 activity and Serpin E1 release [63]. Skin Serpin E1 level is correlated with severity of AD [63]. Cheek injection of Serpin E1 in mice induces itch [63]. This IL-31-BNP-TRPV3 cascade [63] and PAR2-TRPV3-TSLP signaling [106] seem to exhibit a compounded effect to amplify chronic pruritus and cutaneous inflammation in mice (Figure 4). However, their linkage with human itch requires further investigation. Thus far, many therapeutics have been developed toward Serpin-E1-mediated pathways and have made it to clinical trials, but none have been tested for therapeutic use in human itch.

### 4.10. Oncostatin M (OSM)

OSM is released by dermal T cells, macrophages, dendritic cells, neutrophils, and monocytes. Its receptor OSMR resides in sensory neurons expressing BNP; however, OSM does not activate sensory neuronal calcium entry, thus it is different from other pruritogens [187]. OSM knockout or its receptor antagonist reduced itch-like behavior in an inflammatory dermatitis murine model [187]. Intradermal injection of OSM induces itch behavior in mice, albeit via indirect activation of sensory neurons. OSM induces acute itch in mice and promotes keratinocyte G-CSF and IL-8 release [188]. OSM also potentiates histamine- and leukotriene-evoked itch behaviors [187]. Serpin E1 receptor PLAUR resides in *TLR2*^+^ neurons, where Serpin E1 stimulates transcriptional upregulation of *TLR2* and its co-signaling proteins [188]. The PLAUR-TLR2-OSM signaling promotes skin–nerve communication, cutaneous inflammation, and itch, all feeding into an aggravation of AD and exaggerated itch circuits [188]. There are a few neutralizing humanized mAb ongoing clinical trials showing successful proof of principle in murine models for systemic sclerosis and asthma, which might be useful for itch treatment [189].

### 4.11. C-X-C Motif Chemokine Ligand 10 (CXCL10)

CXCL10 is released by diverse immune cells including neutrophils, myeloid cells, and skin keratinocytes. Neutrophil-derived CXCL10 plays a critical role in AD [190]. Although cheek injection of CXCL10 failed to directly cause scratching-like behaviors within a specific dose range [20], CXCL10 has been shown to drive acute itch in a model of allergic contact dermatitis via C-X-C chemokine receptor 3 (CXCR3) signaling in sensory neurons [190]. CXCL10 promotes the recruitment of T cells and dendritic cells (DC) via the receptor CXCR3 [20]. CXCR3 antagonists can alleviate chronic itching [190].

### 4.12. Chemokine C-C Motif Chemokine 2 (CCL2)

Another CCL2 is also implicated in murine allergic contact dermatitis (ACD) [191]. A CCL2 known as monocyte chemoattractant protein 1 (MCP-1) has a receptor called CCR2, which is involved in the pathophysiology of ACD [21]. Intradermal injection of CCL2 into the site of CHS on the cheek evoked site-directed itch- and pain-like behaviors, which are attenuated by prior delivery of an antagonist of CCR2. The targeted CCL2/CCR2 signals may be helpful in the treatment of itch and pain sensations in ACD patients.

## 5. Research Progress on Itch Therapeutical Development

Although substantial research has been conducted to fill the gaps pertaining to the mystery of itching, this debilitating clinical condition is still faced with a highly inapt medical need for efficient treatment. No neuropeptide-targeting therapeutic or agonist/antagonist achieved has successful chronic itch treatment in humans. Many humanized antibodies approved for itch-related diseases have great potential in chronic itch treatment. However, this closely depends on the disease phenotypes and dominance of inflammatory pathways. The combination of different biological agents has resulted in significant advances in the efficacy in some diseases, opening new avenues for future treatment on diseases which are refectory to single treatment. Here, a detailed summary of current development of anti-itch therapeutics is listed in Table 1.

Although pruritus is not the main target in the approval of dupilumab for the treatment of AD [230], the recognition of the high antipruritic effect of this therapy has highlighted the significance of this symptom [231,232,233]. Several small molecule therapeutics have shown promising outcomes by reducing pruritus related with AD and Prurigo Nodularis, such as Janus kinase inhibitor ruxolitnib, and phosphodiesterase 4 inhibitor crisaborole [234,235]. Thalidomide is a drug with anti-inflammatory properties by modification of immune systems. The exact mechanism of action of thalidomide is unknown, but it inhibits TNF-α, IL-6, interleukin-10 (IL-10), interleukin-12 (IL-12), and other pro-inflammatory cytokines. It also modulates natural killer cell cytotoxicity and inhibits NF-κB and COX-2 activity. Erythropoietin (EPO), a hormone produced by the kidneys that stimulates the production of red blood cells, may have some anti-itch properties, as it is shown to reduce plasma histamine concentrations [236].

## 6. Concluding Remarks

Chronic itch is modulated by a complex network involving diverse cell types and systems, and is classified into dermatologic, systemic, neurologic, psychogenic, mixed, and other pruritus. It ranks at the top position of all dermatological diseases. The quality of life of affected individuals is dramatically reduced, as symptoms such as the itching–scratch cycle are combined with a social stigmatization. All chronic itch diseases can show a life quality that is diminished to a degree comparable to diabetes, cancer, or serious cardiovascular events. The most important contribution to this high disease burden comes from inflammatory skin diseases such as psoriasis and AD, two distinct diseases differing in the characteristics of immune response, inflammation, and pathogenesis [237]. However, they have comparable itch scores, such as the itch-specific patient-reported outcome measure used to assess quality of life in patients with chronic pruritus (ItchyQoL) [238], whereas the most common cause of itching among elder patients is xerosis (dry skin). These diseases are very frequent, with a prevalence of 2–4% in the adult population in industrialized countries. Among children, the prevalence of AD is high, but certain chronic itch-associated systemic diseases are more common in older patients, such as chronic kidney disease, hepatic dysfunction, endocrine disorders, and some very heterogeneous diseases that are associated with multiple pruritogenic pathways and pruritogens. Much effort has been made in the scientific community in the last two decades to identify key molecules and biomarkers of those chronic diseases, and a lot of progress has been achieved in psoriasis and AD. Numerous specific and costly therapies are available, which are opposed by the lack of biomarkers to predict the therapeutic outcome at an individual patient’s level and for different categories, including itch with lesion, itch without lesion, and itch on secondary skin lesions. Given the fact that even the best therapies have a non-responder rate between 10% and 20%, identification of biomarkers predicting a response is of great socioeconomic impact for our society. Typically, trials investigating itch treatments are single-center studies with small numbers and often have significant selection bias or conflicting results. Past meta-analyses had insufficient data to recommend one treatment compared with another, and further rigorous trials were needed. A single antagonist is not sufficient due to the switch from one phenotype to another, such as long-term dupilumab treatment switches from T_H_2 to T_H_17, and thus an IL-17 antagonist must be followed. Likewise, treatment with a psoriatic drug can improve psoriasis but cause atopic dermatitis, depending on the individual. Dose optimization and combination of different drugs are required. Therefore, it is important that a modern systematic assessment of the existing evidence be conducted to summarize the effects of current studies. Research into the enhanced understanding of the mechanisms of itch are ongoing and studies identifying novel mechanisms of pruritis in animal and human models are continuing, with the goal to improve the current development of potential therapeutics for chronic itch cases.

## Figures and Tables

**Table 1:** Current advanced therapeutical development for chronic itch.

Drug Category	Drug Names	Advantages	Disadvantages
Targeted monoclonal antibodies (mAb)	Dupilumab	Is a fully human mAb against IL-4Rα that inhibits both IL-4 and IL-13 signaling; is the first approved mAb for AD treatment; demonstrated efficacy and acceptable safety on patients with AD and some other chronic pruritic diseases [192,193]; currently, is ongoing second phase 3 trials on Prurigo Nodularis; is effective in Netherton syndrome, an itchy disease, in a case study [194].	High cost and side effects that cause eye discomfort (especially conjunctivitis) [80,195,196]; administered subcutaneously twice weekly which is painful for children [197]; cannot treat sub-population of patients.
	Tralokinumab	Is a fully human mAb that potently and specifically neutralizes IL-13; in phase 3 for moderate-to-severe adult AD; subcutaneous tralokinumab has an acceptable safety and tolerability profile and appears to provide early improvements in disease symptoms including itch, in participants with moderate-to-severe AD [2]; less costly than dupilumab.	Less effective than Dupilumab [198].
	Lebrikizumab	Is a novel, high-affinity, monoclonal antibody targeting IL-13 that selectively inhibits IL-13 signaling [144,199]; in phase 2 for moderate-to-severe AD; significantly improves clinical manifestations of AD, pruritus, and quality of life in a rapid, dose-dependent manner; generally well tolerated [200,201]; might simultaneously target both inflammation and itch via blocking signals on both immune cells and neurons; less frequency in subcutaneous injection comparing to dupilumab [202].	Might induce conjunctivitis in a few patients with AD [199,203].
	Nemolizumab (CIM331)	Is a humanized antibody against IL-31RA, in the treatment of AD [204]; significantly improves pruritus in patients with moderate-to-severe AD; in two phase 3 trials, nemolizumab plus topical agents improved atopic AD and moderate-to-severe pruritus for up to 68 weeks, without safety issue [205].	Subcutaneous injection might be associated with higher incidence of injection-site reaction than placebo [204].
	Vixarelimab (KPL-716)	Is an OSMRβ antagonist and a fully-human antibody, inhibits the IL-31 signaling and OSM pathway by antagonizing the OSM beta receptor [206]; in phase 2a, subcutaneous injection improves Prurigo Nodularis signs and symptoms, with an average pruritus reduction of 70% by week 8 of treatment as well as significantly improved nodules as early as week 4; safe; currently, it just completed phase 2b in Prurigo Nodularis (ClinicalTrials.gov Identifier: NCT03816891).	No severe adverse effects [206].
	Tezepelumab (AMG-157/ MEDI9929)	Is a human anti-TSLP antibody that prevents TSLP-TSLPR interactions; has high curative effect, good safety, and high tolerance level [207]; in phase 2a AD treatment (ClinicalTrials.gov Identifier: NCT02525094), tezepelumab achieved improvement on week 12 and 16 (post hoc), albeit not statistically significant over placebo and the itch relief is limited [177].	The treatment cycle is longer and expensive, and a few (5.4%) patients developed injection-site erythema, which was not seen in placebo group [177].
	Brodalumab (AMG 827)	Is a human anti–IL-17 receptor A IgG2 mAb; in phase 3, it significantly and rapidly improves moderate-to-severe psoriasis, including itch, in patients [208,209,210]; approved by FDA to treat adult moderate-to-severe plaque psoriasis.	Subcutaneous injection might be associated with higher incidence of injection-site reaction than placebo [204].
	Secukinumab	Is a fully human anti-interleukin-17A IgG1 monoclonal antibody [211]; is well-tolerated, safe, and effective in psoriasis and associated itch and pain; approved by the US FDA and European Medicines Agency for moderate-to-severe plaque psoriasis and psoriatic arthritis [212,213]; in phase 2 for AD treatment including intrinsic, Asian, and pediatric AD, secukinumab is not effective in reduction of epidermal thickness, epidermal hyperplasia, and immune cell infiltrates, or inflammatory markers in relation to TH17/IL-23 at week 16 [212].	Observed adverse events, all in secukinumab-treated patients: orbital cellulitis, upper respiratory infection, and streptococcal pharyngitis [212]; treatment of AD is not effective, however, may be helpful in conjunction with T_H_-2 biological agents [212].
	Ixekizumab	Is an IgG4 monoclonal antibody that targets IL-17A; achieved outstanding performance in the itch and moderate-to-severe psoriasis treatment effect at 12 weeks [214]; can demonstrate persistent efficacy through 108 weeks (80 mg ixekizumab every 2 weeks up to week 12 and every 4 weeks thereafter); FDA approved for treatment of adult moderate to severe plaque psoriasis, active psoriatic arthritis [215,216].	Mild or moderate adverse events included nasopharyngitis, upper respiratory tract infections, injection-site reactions, arthralgia, bronchitis, and headache [217]; some patients may be associated with eczematous eruptions in the face [218].
	Ustekinumab	Is an IL-12/IL-23p40 IgG1κ monoclonal antibody that suppresses Th1, Th17, and Th22 activation; approved for psoriasis patients [219]; beneficial clinical effects in moderate-to-severe AD patients [220]; has unique mechanistic effects in AD as early as 4 weeks of treatment; strongest anti-inflammatory effects already occur within 4–8 weeks following an ustekinumab dose, with waning efficacy thereafter [221].	Individual patients were excluded from analyses after week 28 due to newly developed contact dermatitis and due to worsening skin infection (eczema herpeticum) [221].
	Risankizumab	Is a novel IL-23 mAb, with relatively high efficacy and low risk; had been approved by the FDA in April 2019 to treat AD [214], and in June 2022 to treat moderate-to-severe active Crohn’s disease in adults, an itchy disease.	The most relevant adverse events were nasopharyngitis, upper respiratory tract inflammation, and injection site reaction [214].
	Guselkumab	Is an IL-23 mAb; approved for moderate-to-severe plaque psoriasis. It has demonstrated safety and efficacy in phase III clinical trials [222,223,224,225]; combined treatment by dupilumab and guselkumab rapidly and sustainably improved itch, erythroderma, and eczema in severe AD associated with congenital ichthyosiform erythroderma (CIE), whereas treatment with dupilumab or guselkumab was less or not effective [226].	There are scarce data regarding its drug survival in clinical practice [224]; serious adverse events included serious infections, nonmelanoma skin cancer, malignancies other than nonmelanoma skin cancer, and major adverse cardiovascular events [222].
	Tildrakizumab	Is a high-affinity, humanized, IgG1 κ antibody targeting p19 subunit of IL-23; demonstrated superior efficacy, safety, and long-term control of moderate-to-severe chronic plaque psoriasis; FDA-approved in 2018 for moderate-to-severe plaque psoriasis [214,227].	Caused some minor adverse events, including body aches or pain, chills, cough, difficulty in breathing, ear congestion, fever, etc. [227].
IgE antibody	Omizumab	Is a humanized IgG1 mAb. It binds to the Ce3 domain of IgE with higher affinity; is highly selective for human and non-human primate IgE, with higher efficacy, good safety and high tolerance level in vivo; effective in AD, bullous pemphigoid, and urticaria [228]; received FDA breakthrough therapy designation for patients with chronic spontaneous urticaria in patients that cannot be treated effectively by H1 antihistamine.	Reported serious adverse events, viral upper respiratory tract infection (20%), injection-site reaction [229].

## Data Availability

Not applicable.
